# The importance of microglia in the development of the vasculature in the central nervous system

**DOI:** 10.1186/2045-824X-5-4

**Published:** 2013-02-19

**Authors:** Tom Arnold, Christer Betsholtz

**Affiliations:** 1 Departments of Pediatrics and Anesthesiology and Perioperative Care, University of California San Francisco, 95158, San Francisco, CA, USA; 2 Department of Medical Biochemistry and Biophysics, Division of Vascular Biology, Karolinska Institutet, 17177, Stockholm, Sweden; 3 Department of Immunology, Genetics and Pathology, Rudbeck Laboratory, Uppsala University, Dag Hammarskjölds väg 20, 75185, Uppsala, Sweden

## Abstract

The body’s vascular system is thought to have developed in order to supply oxygen and nutrients to cells beyond the reach of simple diffusion. Hence, relative hypoxia in the growing central nervous system (CNS) is a major driving force for the ingression and refinement of the complex vascular bed that serves it. However, even before the establishment of this CNS vascular system, CNS-specific macrophages (microglia) migrate into the brain. Recent studies in mice point to the fundamental importance of microglia in shaping CNS vasculature during development, and re-shaping these vessels during pathological insults. In this review, we discuss the origin of CNS microglia and their localization within the brain based on data obtained in mice. We then review evidence supporting a functional role of these microglia in developmental angiogenesis. Although pathologic processes such as CNS ischemia may subvert the developmental functions of microglia/macrophages with significant effects on brain neo-angiogenesis, we have left this topic to other recent reviews (Nat Rev Immunol 9:259–270, 2009 and Trends Mol Med 17:743–752, 2011).

## Microglia – specialized macrophages of the CNS

Microglia are specialized macrophages of the central nervous system involved in immune regulation, tissue development, homeostasis and wound repair. Microglia were first observed by Virchow in the mid-nineteenth century (see), and described in greater detail by Pio del Rio-Hortega in 1932. In this almost prescient work, del Rio-Hortega described microglia morphology, plasticity during development and with pathological insult, their cellular origin, and microglia association with white matter tracts and blood vessels. Despite an immense amount of research on microglia origin and function since then, these early views remain surprisingly accurate.

### Microglia derive from primitive yolk sac macrophages

Microglia belong to the mononuclear phagocytic system - a family of cells that includes committed precursors in the bone marrow, circulating blood monocytes and tissue macrophages in every organ of the body including the CNS. Mononuclear phagocytes are typified by their ability to ingest large particles; their morphology; their expression of common surface markers including CD11b, CD68, Colony Stimulating Factor 1 Receptor (CSF1R), chemokine receptor CXCR3, and plasma membrane glycoprotein F4/80 [1]; and their presumed hematopoietic origin. While microglia certainly meet the functional and morphological definition of a mononuclear phagocyte [2-4], their developmental origin has until recently been less clear.

**Figure 1:**
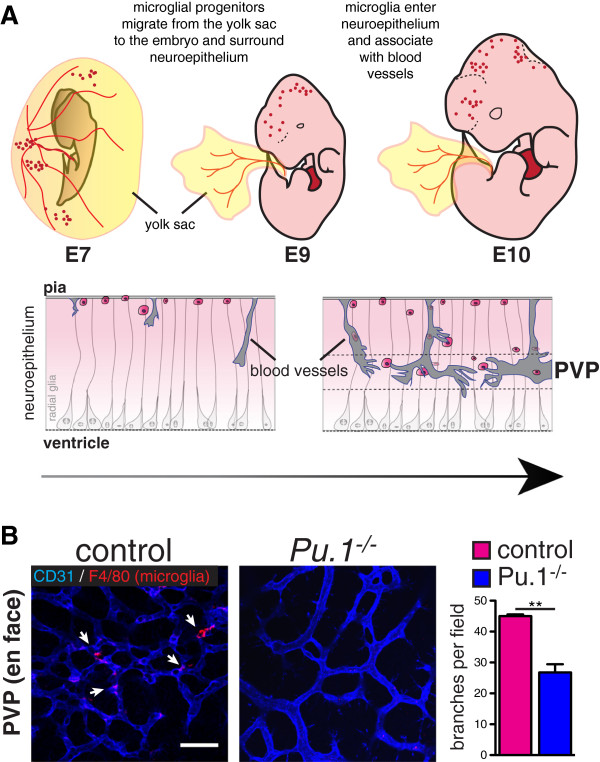
**A) Microglia originate from myeloid precursors in the yolk sac, which migrate into the neuroepithelium by E10.** They associate with radial glia and with blood vessels (also ingressing into the brain from the pial surface) where they may promote fusion of vascular tip cells in the periventricular vascular plexus (PVP). Arrow indicates progressive development from mouse embryonic day (E) 7 to E10. (Modified from [19].) **B**) Bottom: Reduced vascular branching in the brains of microglia-deficient *Pu.1*^−/−^ mouse embryos. Flat mounted brains from embryonic day 12 mice stained for endothelium (CD31, blue) and microglia (F4/80, red). Branch points quantified from 4 mutants and controls. Students T Test P < 0.005.

In mice, hematopoietic stem cells (HSCs) emerge from the dorsal aorto-gonado-mesonephros (AGM) region 10.5 days after conception (embryonic day (E) 10.5), then migrate to the fetal liver where they expand and differentiate before definitive hematopoiesis in the spleen and bone marrow [5-8]. In adult mice, blood monocytes, classical dendritic cells, and certain tissue macrophages derive from, and are continuously replaced by, bone marrow-derived HSCs. It was previously thought that microglia arose from hematopoietic precursors in two waves of recruitment and differentiation [9,10]. However, it is now clear, based on evidence from bird, fish and mammals, that yolk-sac derived macrophage precursors contribute significantly, if not entirely, to the brain’s microglia. In avian embryos, analyses using chick-quail transplantation and parabiosis chimeras show that yolk sac-derived macrophages migrate to and invade the CNS through the pial basal lamina before and independent of CNS vacularization [11,12]. Subsequent live recordings of cell movements in zebrafish embryos revealed that yolk sac-derived macrophages migrate through the cephalic mesenchyme before its vascularization to reach the brain pial surface and the roof of the 4th ventricle, from where they subsequently invade the neuroepithelium and eventually acquire microglial characteristics [13]. Recently, fate mapping studies in the mouse using genetic lines such as *Cx3cr1*-green fluorescent protein (GFP), *Csf1r-GFP*, and cell surface markers, show that microglial precursors originate in the yolk-sac, colonize the surface of the brain between E9.5 and E10.5 (i.e. before the onset of HSC formation in the AGM region), then appear within the brain neuroepithelium in association with blood vessels by E10.5 (Figure 1) [14-17]. Lineage analysis with tamoxifen-inducible *Runx1-CreERtm*[14] or *Csf1r-CreERtm* mice [15] suggests that the first wave of yolk-sac derived microglia is specified before E8.0. Microglia proliferate throughout embryogenesis and self-renew without significant contribution from the bone marrow in the steady state [14,15,18]. While bone-marrow derived monocytes may infiltrate the brain parenchyma in conjunction with irradiation or inflammation [19], these cells later disappear, and do not significantly contribute to the population of resident microglia [18].

The yolk sac origin of microglia is further supported by elegant experiments done in mice lacking the transcription factors PU.1 [15,20-22] or Myb [15], and in mice with inactivated *Csf1* (*CSF1 op/op* mice) [23], or *Csf1r* genes [24,25]. PU.1 acts in part by activating transcription of *Csf1r*[26], which is highly expressed in macrophages and microglia during development and throughout adult life [14]. The major ligands of CSF1R, IL34 and CSF1, are highly expressed in the brain parenchyma during microglial colonization [27]. Mice with genetic deletion of *Pu.1*[15] or *Csf1r*[14] have substantially reduced yolk sac macrophages and microglia, but normal numbers of circulating monocytes [14,15]. *Csf1 op/op* mice have a milder reduction in microglia, consistent with an important role for IL34 in microglial homeostasis [24,27]. Further work with Myb-deficient mice clarified the distinct lineage of microglia. Genetic loss of Myb blocks the generation of HSCs and their progeny (including circulating monocytes and granulocytes), but these mice have normal numbers of tissue macrophages and microglial cells [15]. Similarly, myeloid-specific expression of diphtheria toxin in *LysMCre;Rosa26DTA* transgenic mice eliminates monocyte-derived macrophages without effect on resident microglia [16]. Taken together, these studies indicate that the large majority of embryonic and adult brain microglia are derived from early yolk sac precursors.

### Patterns of brain colonization by microglia

Once born, yolk-sac derived macrophage precursors migrate into and colonize the whole (mouse) embryo between E9.5 and E10.5 (Figure 1) [14,15]. The first organ to be colonized is the brain. Subsequent phases of microglial brain colonization follow a stereotyped pattern (see [28]). Microglia invade the brain through the pial surface, then migrate and proliferate, populating the brain in a dorsal-to-ventral and rostral-to-caudal gradient. During this time, microglia associate with radial glia and blood vessels, and are found in close proximity to dying cells. Eventually, microglia are notably excluded from the neuroepithelium and cortical plate, and then are widely distributed in the adult brain, except in areas of densely packed neuron cell bodies such as the pyramidal cell layer. The early association of microglia around blood vessels has led to the hypothesis that microglia may enter the brain through the developing vasculature. Indeed, E10.5 *Ncx1*^*−/−*^ embryos [29], which lack heartbeat and blood flow, are devoid of microglia [14]. However, it is not clear whether lack of blood flow *per se* disrupts brain vascular development in these mutants (they may lack brain vasculature altogether). It is possible that some microglia migrate along the abluminal surface of pial penetrating vessels, independent of blood flow, or that a small population of microglia (not detected by flow cytometry in [14] initially invades the brain without contact with vessels [16,30]. Notably, microglia populate the embryonic retina well before post-natal retinal angiogenesis occurs [16,31,32], and as mentioned above appear to colonize zebrafish and chick brains before CNS vascular invasion [11-13]. Shortly after entering the neuroepithelium, microglia associate with the developing blood vessels in the brain and retina [11-13,17,33], and microglia and vessels may therefore directly influence each other’s development, an idea that lends support from ex vivo studies [32].

## Microglia shape the developing CNS vasculature

Because the developing CNS lacks intrinsic vasculature, CNS blood vessel development occurs exclusively via angiogenesis [34-36]. Attracted by proangiogenic signals, new capillaries sprout from perineural vessels, and invade the neuroectoderm around E10 in mice. These nascent capillaries are composed of tip cells at the vascular front, followed by proliferative stalk cells. Tip cells extend filopodia toward guidance cues such as VEGF-A. VEGF-A induces expression of the Notch ligand, Dll4, predominately in tip cells. Dll4 then activates Notch in adjacent cells, which down-regulates VEGF receptors and up-regulates angio-suppressive factors like sFlt1 and Jagged-1, promoting a stalk cell phenotype (reviewed in [36]). The interplay between VEGF and Notch signaling is highly regulated with additional inputs from other major signaling pathways including BMPs [37,38], Semaphorins [39], and Wnt/βcatenin [40,41]. Additional signaling pathways that regulate tip cell formation and sprouting include sphingosine-1-phosphate and its receptor S1pr1 [42-44]. During vascular sprouting, tip cells anastomose with neighboring tip cells, creating vascular loops. In this way, vessels sprout, extend, branch and anastomose, iteratively, toward the center of the neural tube where they establish a temporary plexus, termed the periventricular vascular plexus (PVP), around the CNS ventricular spaces and spinal cord’s central canal [45-47]. As the CNS grows and differentiates, these vessels associate with microglia, pericytes, neuroepithelial radial glia and neuroblasts, and later astrocytes; CNS vessels are refined, arteries and veins are established, and the mature neurovascular system takes form.

The retina and optic nerve represent highly specialized extensions of the forebrain. While its vascularization occurs by angiogenic sprouting similar to the brain, the timing and scaffolds that guide angiogenesis are partly different [48]. During the first week of life, an astrocytic network arising from the optic nerve invades into the retina in a centrifugal fashion. As the primitive hyaloid (hv) vessels that supported the embryonic eye development regress, a new primary vascular plexus extends into the retina, following structural and morphogenic cues provided by astrocytes and Müller glia. At 7 to 9 days of postnatal life, vessels sprout perpendicularly into deeper layers of the retina, forming a deep vascular plexus in the outer plexiform retinal layer (OPL). During the next 3 weeks, retinal vasculature continues to sprout, remodel and differentiate into arteries and veins and a mature neurovascular network is established by 6 weeks of age.

### Microglia influence CNS vascular development

As discussed above, microglia migrate into the CNS and retinal neuroepithelium before vessels do. Microglia are therefore uniquely positioned to influence the early sprouting, migration, anastomosis, and refinement of the growing CNS and retinal vascular systems. Studies of angiogenesis after microglial depletion, or in mice lacking microglia, strongly support this concept. Checchin et al. [49] administered clodronate liposomes either systemically to deplete macrophages and circulating monocytes, or intravitreally, which depleted retinal microglia without reducing circulating monocytes. These authors found that reducing retinal microglia numbers was associated with a decrease in retinal vascular density. Selective depletion of circulating monocytes had no impact on retinal blood vessels. Importantly, intravitreal co-administration of microglia with clodronate restored vascularity of the developing retina, suggesting a microglia-specific effect on retinal vascular development. Similarly, Kubota et al. [33] found that *Csf1 op/op* mice, which initially lack retinal microglia, have a significant decrease in branching of the primary vascular plexus. Also, intra-vitreal administration of CSF1-neutralizing antibodies, or systemic administration of a CSF1R kinase inhibitor, decreased microglia numbers with commensurate reductions in vascular branching. Branching in these mice recovered as development progressed, suggesting that microglia principally effect developmental vascular remodeling, but do not contribute to maintenance of adult vascular patterns. Interestingly, they found that *Csf1 op/op* mice, and mice with pharmacologic microglial depletion, have comparatively normal numbers of endothelial tip cells and filopodia. This suggests that microglia facilitate branching anastomosis, but not tip cell extension. In contrast, Unoki et al [50] found that depletion of microglia using clodronate liposomes increased VEGF-mediated neovascular sprouts in an *ex vivo* retina culture model.

The concept that microglia may act to “bridge” vascular sprouts during CNS vascular development was introduced by the work of Fantin et al. [16]. This group studied vascular ingression and branching in embryonic hindbrains and retinas of mice lacking macrophages and microglia (*Pu.1*^*−/−*^ and *Csf1 op/op* mice). In the hindbrain, microglia numbers were correlated with numbers of branch points, and were frequently found to be in contact with neighboring endothelial sprouts. Loss of microglia was associated with a significant decrease in the numbers of vascular branch points in the brain periventricular vascular plexus, without significant effects on the numbers of tip cells, filopodia, or radial vessels ingressing from the surface of the brain (see also Figure 1). Selective depletion of circulating monocytes using *LysmCre;Rosa26DTA* transgenic mice did not affect hindbrain vascular branching, again suggesting that resident microglia are principally responsible for facilitated branching. As VEGF is central to tip cell guidance and vascular branching, and VEGF functions as a macrophage chemoattractant in tumors and *in vitro*, the authors evaluated VEGF mRNA levels in the hindbrains of microglia-deficient embryos and found no difference. They also analyzed microglia numbers and branching patterns in mice lacking VEGF, or with selective expression of VEGF120 (*vegfa*^*120/120*^ mice, which lack heparin binding VEGF isoforms while retaining the diffusible VEGF120 isoform). These mutant mice revealed abnormalities in hindbrain angiogenesis distinct from those observed in microglia-deficient mice: *vegfa*^*120/120*^ mice had more global vascular deficiencies, including decreases in the numbers of tip cells and penetrating radial vessels, and *vegfa 120/120* and *vegfa*^*flox/+*^*;NesCre* mice had a more pronounced reduction in vascular branching than microglia-deficient mice. Microglia numbers were unaffected in these VEGF mutants. These results imply that microglia are not a significant source of VEGF, that VEGF (unlike CSF1 or IL34) is not a major chemoattractant or survival cue for microglia, and that the mechanisms of microglia-facilitated branching may be distinct from, and complimentary to, VEGF-mediated sprouting.

A recent report from our laboratory confirmed and extended many of these observations [32]. We found that microglia-deficient mice (*Csf1 op/op* mutants and *Pu.1* knockouts) had reduced numbers of vascular branch points in the retina, and that the angle of filopodia extending from tip cells was reduced in these mice. We used microglia co-cultured in collagen matrix with mouse aortic rings to study microglia-vascular interactions more deeply. These studies revealed an apparent two-way communication between aortic rings and microglia: aortic rings induced the migration of microglia towards the ring, while microglia significantly increased vascular branches emanating from the ring. Interestingly, media from cultured microglia added separately to cultured aortic rings had a similar, albeit less potent, effect on vascular branching. This suggests microglia-blood vessel contact enhances, but is not necessary to induce branching, and that microglia may release soluble factors that stimulate sprouting/branching. Addition of soluble Flt1 (a VEGFR1 ectodomain that traps and neutralizes VEGFA, VEGFB and, placenta growth factor (PIGF) or VEGFR1 neutralizing antibody to microglia-aortic ring cultures did not effectively inhibit microglia-induced branching, suggesting that VEGFA and sFlt1 are not major microglia-derived factors responsible for branching induction.

In contrast to these findings, Stefater et al. [51] recently uncovered a mechanism whereby microglia may *suppress* angiogenic branching through a Wnt-Flt1 pathway. Here, they found that microglia associated with the deep retinal vascular plexus specifically express various Wnt signaling components including Wnt5a and Wnt11 ligands, and Wnt receptors Fzd7, Fzd8 and Lrp5. Microglia-specific haploinsufficiency of the common Wnt-ligand transporter, Wls (*Wls*^*flox/+*^*;Csf1rCre* mice), resulted in increased vascular branching in the deep vascular plexus. Similarly, Wnt5a and Wnt11 haploinsufficiency resulted in a similar phenotype. Interestingly, deletion of Lrp5 from microglia had the opposite effect, with a significant reduction in vascular branching. They found that Wnt5a induced expression of sFlt1, that *Wls*^*flox/+*^*;Csf1rCre* mice have reduced expression of sFlt1, and that microglia-specific haploinsufficiency of Flt1 phenocopies the Wls and Wnt5a and Wnt11 mutants. Taken together, their results indicate that microglia can suppress vascular branching in the deep retinal vascular plexus by secreting Wnt ligands, which induce, in an autocrine fashion, secretion of the VEGF inhibitory protein, sFlt1. These results appear to contrast with those reported by Fantin et al., who found no difference in branch density in the deep retinal vascular plexus in the absence of microglia [13], which they explained was the net outcome of less branching and less pruning. This result pinpoints the potential complex role of microglial cells in retinal angiogenesis, as regulators of endothelial sprouting, branch fusion and regression.

The feedback loop between VEGF and Notch involves regulation of both VEGFR-2 and VEGFR-3, although the individual contribution of each of these VEGF receptors remains unclear [41,52-54]. The primary ligands for VEGFR-3, VEGF-C and VEGF-D, are highly expressed by microglia, and VEGF-C-positive microglia are found near the fusion points of VEGFR-3-positive vascular sprouts [54]. While *Vegfd* knockout mice have no apparent retinal vascular phenotype, *Vegfc* heterozygous mice display delayed retinal vascularization and decreased branching, but increased vessel sprouting and filopodia [54]. Interestingly, this phenotype is different from mice with loss of function deletions or antibody blockade of VEGFR-3 [52,53]. There are alternative explanations for these discrepancies. One group proposes that VEGFR-3 has both ligand-dependent (and pro-angiogenic) and ligand-independent (anti-angiogenic) signaling activities [54]. Another group suggests that ligand-independent signaling by VEGFR-3 is pro-angiogenic (when Notch signaling is suppressed) [52]. Further experimentation should clarify the roles of microglia-derived VEGF-C/D-VEGFR3 signaling in vascular development.

Other groups have recently explored potential roles for Notch signaling in microglia-endothelial cell interactions. Outtz et al. [55] found that Notch signaling is activated in retinal microglia, which are closely associated with endothelial tip cells expressing the Notch ligand Dll4. Moreover, genetic deletion of Notch1 in retinal microglia led to a subtle reduction in the numbers of microglia found at the vascular front. Interestingly, Hoffman et al. [56] found that the Notch ligand, Jagged1, is highly expressed in perivascular cells of the retina, including microglia. Further studies should evaluate the specific roles of Notch signaling in microglia, and the impact of this signaling on retinal vascular development.

## Outlook

The development of organ-specific vascular beds is dependent upon the close communication between the vascular cells (endothelial cells and mural cells) on the one hand, and resident cells of the organ, on the other. The CNS vasculature is in many ways unique in its anatomy and regulation, and it harbors a highly specific barrier – the blood–brain, or blood-retina, barrier. The development of the CNS vasculature hence occurs in tight association with the development of other components of the CNS, and as a result of reciprocal communication between the endothelial cells and different emerging CNS cell types. This review focuses on the role of microglial cells – a CNS-specific type of macrophage – and also mentions in passing the importance of other cells types, such as radial glial cells and astrocytes, as sources of VEGFs, Wnts and other signaling molecules that control the shape and function of the emerging vasculature. Although recent studies provide compelling evidence for a role of microglia in shaping the nascent vascular plexuses in the brain and retina, the molecular mechanisms remain to be elucidated, and the possibility remains that microglia play a different role at different locations, for example in the different retinal capillary plexuses. It also remains to be shown what role, if any, microglia play in vascular homoeostasis in adult physiological and pathophysiological processes. Microglial cells become activated in conjunction with pathological insults and disruption of the blood–brain barrier, where they may play protective or pathogenic roles. These data are not discussed in the present review, which is focused on physiological development. However, the awareness of microglia as a unique CNS cell type with a distinct ontogeny and equipped with specific developmental and pathological functions compared to other glial cell types, has recently increased. Our perspectives on these cells will undoubtedly grow rapidly in the coming years.

## Ethical approval

Animal experiments were approved by the Stockholm’s North Ethical Committee for Animal Research.

## Competing interests

The authors declare that they have no competing interests.
